# A SISCAPA-based approach for detection of SARS-CoV-2 viral antigens from clinical samples

**DOI:** 10.1186/s12014-021-09331-z

**Published:** 2021-10-22

**Authors:** Kiran K. Mangalaparthi, Sandip Chavan, Anil K. Madugundu, Santosh Renuse, Patrick M. Vanderboom, Anthony D. Maus, Jennifer Kemp, Benjamin R. Kipp, Stefan K. Grebe, Ravinder J. Singh, Akhilesh Pandey

**Affiliations:** 1 Department of Laboratory Medicine and Pathology, Division of Clinical Biochemistry and Immunology, Mayo Clinic, Rochester, MN 55905 USA; 2 Manipal Academy of Higher Education, Manipal, 576104 Karnataka India; 3 Institute of Bioinformatics, International Technology Park, Bangalore, 560066 Karnataka India; 4 Center for Molecular Medicine, National Institute of Mental Health and Neurosciences, Hosur Road, Bangalore, 560029 Karnataka India; 5 Center for Individualized Medicine, Mayo Clinic, Rochester, MN 55905 USA; 6 Department of Laboratory Medicine and Pathology, Division of Laboratory Genetics and Genomics, Mayo Clinic, Rochester, MN 55905 USA; 7 Department of Medicine, Division of Endocrinology, Mayo Clinic, Rochester, MN 55902 USA

**Keywords:** SARS-CoV-2, COVID-19, SISCAPA, Mass spectrometry, Parallel reaction monitoring (PRM)

## Abstract

SARS-CoV-2, a novel human coronavirus, has created a global disease burden infecting > 100 million humans in just over a year. RT-PCR is currently the predominant method of diagnosing this viral infection although a variety of tests to detect viral antigens have also been developed. In this study, we adopted a SISCAPA-based enrichment approach using anti-peptide antibodies generated against peptides from the nucleocapsid protein of SARS-CoV-2. We developed a targeted workflow in which nasopharyngeal swab samples were digested followed by enrichment of viral peptides using the anti-peptide antibodies and targeted parallel reaction monitoring (PRM) analysis using a high-resolution mass spectrometer. This workflow was applied to 41 RT-PCR-confirmed clinical SARS-CoV-2 positive nasopharyngeal swab samples and 30 negative samples. The workflow employed was highly specific as none of the target peptides were detected in negative samples. Further, the detected peptides showed a positive correlation with the viral loads as measured by RT-PCR Ct values. The SISCAPA-based platform described in the current study can serve as an alternative method for SARS-CoV-2 viral detection and can also be applied for detecting other microbial pathogens directly from clinical samples.

**Supplementary Information:**

The online version contains supplementary material available at 10.1186/s12014-021-09331-z.

## Introduction

The coronavirus outbreak that started at the end of 2019 has become a major challenge to the health system worldwide [1]. The transmission of the SARS-CoV-2 virus from human-to-human is very high necessitating early and accurate diagnosis of the infected individuals for disease control. Reverse transcriptase-polymerase chain reaction (RT-PCR) has been the gold standard testing method for the detection of SARS-CoV-2. Several other methods have been developed for the diagnosis of COVID-19 which are based on the detection of viral proteins, anti-viral antibodies or nucleic acids [2]. Owing to increasing number of infections worldwide, alternate screening methodologies need to be explored especially in light of logistical issues pertaining to reagents and instruments for RT-PCR-based tests [3, 4].

Mass spectrometry is an excellent analytical tool that can be applied for analysis of a diverse array of analytes such as small molecules, proteins and peptides [5]. Targeted analysis using multiple reaction monitoring (MRM) or parallel reaction monitoring (PRM) has become a feasible alternative to immunoassays [6]. Automation for sample preparation and data analysis has further helped in increasing the specificity and throughput. Several groups have attempted to automate the chromatographic conditions and analysis workflow for the detection of SARS-CoV-2 using mass spectrometry. Cardozo, et al. used turbulent flow chromatography coupled to tandem mass spectrometry, which enabled analysis of samples with increased throughput and demonstrated a sensitivity of ~ 78% [7]. Another study used a MALDI-MS approach for the diagnosis of the SARS-CoV-2 based on the distinct spectral features in COVID-19 patients selected by machine learning approaches, however, the chemical identity of the spectral features remains unknown [8]. There are several other published studies which describe detection of viral antigen from clinical specimens based on targeted proteomics [9–13]. Notably, Renuse et al. employed nucleocapsid protein enrichment followed by LC-FAIMS-PRM approach for detection SARS-CoV-2 from nasopharyngeal swab (NP swab) samples. In addition, a machine learning approach was used on fragment ion intensities to achieve a sensitivity of 98% and a specificity of 100% [14].

MRM or PRM assays are based on targeted measurement of peptides that are unique to the protein of interest. However, the sensitivity of these assays can be limited in complex matrices. Enrichment of the specific peptides by affinity purification prior to LC–MS/MS analysis improves the sensitivity and performance of the assay especially in biological fluids such as serum, plasma, cerebrospinal fluid, urine, and whole blood, which are commonly used in clinical laboratories. Stable Isotope Standards and Capture by Anti-Peptide Antibodies (SISCAPA) workflow enables the enrichment of target peptides by employing anti-peptide antibodies [15]. SISCAPA-based target enrichment followed by targeted MS/MS analysis has been shown to yield precise and accurate quantitation of the target analytes. Several studies have used SISCAPA workflow to develop quantitative assays for proteins [16–22]. Developing the SISCAPA workflow for SARS-CoV-2 detection might provide an improvement in sensitivity, especially in clinical samples with low viral load.

In this study, we generated antibodies against viral nucleocapsid-derived peptides and developed a semiautomated yet sensitive SISCAPA-based approach for the detection of virus from nasopharyngeal swab samples from COVID-19 patients. Using this approach, we were able to successfully detect viral peptides directly from patient samples including those with low viral loads.

## Methods

### Sample collection and handling

Residual SARS-CoV-2 positive and negative nasopharyngeal swab samples were collected after routine diagnostic testing using RT-PCR. These samples were collected in sterile phosphate buffer saline solution and were stored at – 80 ℃ until further processing. All the samples were collected after prior approval from Mayo Clinic's Institutional Review Board.

### Selection of target peptides and peptide synthesis

Annotated nucleocapsid protein sequences were downloaded for the SARS-CoV-2, SARS-CoV, MERS and the common human coronaviruses (OC43, HKU1, NL63 and L229E) from the NCBI. Multiple sequence alignment of downloaded nucleocapsid protein sequences was carried out using Clustal Omega at EMBL-EBI [23]. Non-synonymous coding variants identified in the SARS-CoV-2 genomes available from GISAID platform (https://www.gisaid.org/) were downloaded from the GESS database [24]. Peptides encoded in the regions of nucleocapsid protein that were mutated in > 1% of sequenced cases in GISAID were excluded. The peptide standards were purchased from New England Peptide (Gardner, MA) and were handled as per published recommendations [25].

### Processing of nasopharyngeal swab samples

750 µl of each nasopharyngeal swab samples were collected in a 96-well plate. Viral inactivation and reduction of proteins was done by addition of 15 µl of 1 M dithiothreitol and 15 µl of 0.1% Zwittergent 3-16 (Z-3-16) detergent (EMD Millipore, Billerica, MA, USA) and incubating at 70 ℃ for 30 min. Alkylation was done using 45 µl of 1 M iodoacetamide (Sigma, St. Louis, MO, USA), vortexed and incubated in dark for 30 min. Finally 250 µl of 1 M Tris hydrochloride solution (Sigma, St. Louis, MO, USA), pH 8.0 buffer was added and digested using 2.5 µg of Worthington TPCK treated trypsin (Thermo Fisher Scientific, Waltham, MA, USA) by incubating at 37 ℃ for 16 h. The reaction was stopped by adding 5 µg of tosyl-L-lysyl-chloromethane hydrochloride (TLCK) (Millipore Sigma, St. Louis, MO, USA) to each sample.

### Antibody coupling to MSIA tips and process automation

The polyclonal anti-peptide rabbit antibodies (1 µg) were conjugated to custom Mass Spectrometric Immunoassay (MSIA) Tips by Thermo Fisher Scientific (Tempe, AZ). All the samples were processed and digested as described under sample preparation section. The digested samples were then subjected to binding and elution using a Versette PlateMate robotic workstation (Thermo Fisher Scientific, Tempe, AZ). A total of 1 ml of digested sample was used for the enrichment. The digest was split into three parts and peptides were then enriched separately with repeated (1000 repetitions) drawing and expelling of 300 µl aliquot of sample volume through the antibody crosslinked MSIA Tips. After enrichment, target peptides were eluted in 100 µl of 0.2% TFA containing 0.002% of Z3-16 detergent (Millipore Sigma, Burlington, MA). The samples were then dried and analyzed with high-resolution liquid chromatography tandem mass spectrometry (LC–MS/MS) using PRM as described below.

### PRM analysis of NP swab samples processed using SISCAPA workflow

PRM analysis was performed on an Orbitrap Eclipse Tribrid Mass Spectrometer interfaced with an UltiMate 3000 RSLC nano system (Thermo Scientific, San Jose, CA). The peptides were loaded onto a trap column (PepMap C_18_ 2 cm × 100 µm, 100 Å) at a flow rate of 20 µl/min using 0.1% formic acid in water and separated on an analytical column (PepSep 10 cm × 100 µm, C_18_ 1.9 µm, 100 Å, PepSep, Marslev, Denmark) with a flow rate of 500 nl/min with a linear gradient of 5 to 40% solvent B (100% ACN, 0.1% formic acid) over a 25 min gradient. Both precursor and fragment ions were acquired in the Orbitrap mass analyzer. Precursor ions were acquired in *m/z* range of 350–1700 with a resolution of 120,000 (at *m/z* 200), AGC target of 3 × 10^4^, maximum injection time of 200 ms and isolation window of *m/z* 1.6. Precursor fragmentation was carried out using higher-energy collisional dissociation method using 28% normalized collision energy. The MS/MS spectra were acquired at a resolution of 30,000 (at *m/z* 200) in the orbitrap analyzer. The scans were arranged in top-speed method with 3 s cycle time between MS and MS/MS. Ion transfer capillary voltage was maintained at 2.2 kV. For internal mass calibration, lock mass option was enabled with polysiloxane ion (*m/z*, 445.120025) from ambient air.

### Data analysis

The data analysis was performed using Skyline [26]. The peaks were manually verified for correct detection of the peak, exact integration and lacking the interferences. The total peak area was calculated by summing all the selected transitions. The coefficient of variation (CV) was calculated by dividing standard deviation with mean and expressed as a percentage.

## Results and discussion

### Selection of candidate peptides for the detection of SARS-CoV-2 using SISCAPA approach

The SISCAPA approach employs immunoprecipitation of peptides as surrogates for protein quantitation. Thus, selection of peptides unique to the target protein of interest is a crucial step for the development of targeted method. The nucleocapsid protein is the most abundant protein in the SARS-CoV-2 proteome (~ 1000 copies/virion). Owing to its high abundance, it is being used for the development of assays using various approaches for the detection of SARS-CoV-2 virus in clinical samples. However, due to its interaction with various cellular proteins during viral replication, immunoprecipitation of nucleocapsid protein could lead to increase in the background matrix complexity and thus decrease in the sensitivity of the targeted method. Thus, enrichment of peptides from digested samples using anti-peptides antibodies could alleviate these issues increasing the sensitivity of the targeted assay.

**Fig. 1:**
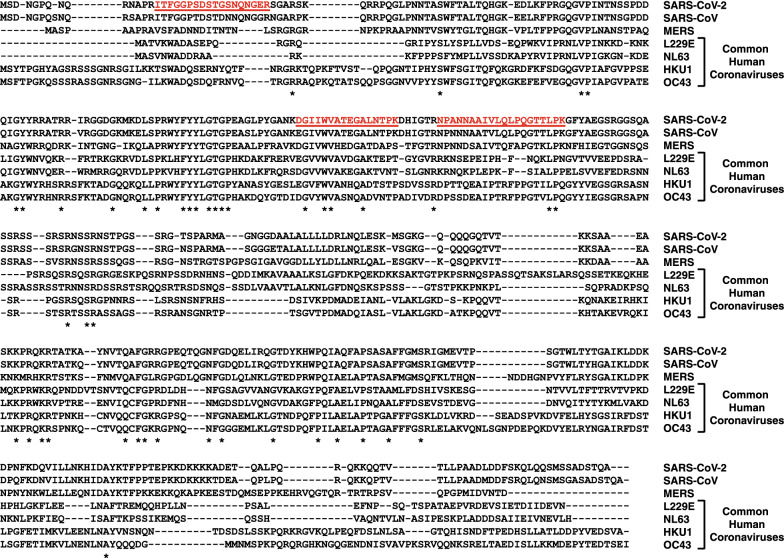
Sequence alignment of SARS-CoV-2 nucleocapsid protein against related coronaviruses. The nucleocapsid protein sequence of SARS-CoV-2 virus was aligned with that of SARS-CoV, MERS and common human coronaviruses (L229E, NL63, HKU1 and OC43). The tryptic peptide sequences highlighted in red indicate the three proteotypic peptides selected for generation of anti-peptide antibodies as they are unique to SARS-CoV-2

As SARS-CoV-2 belongs to the family of coronaviruses which also include common cold viruses, peptides unique to SARS-CoV-2 were selected by performing multiple sequence alignment of nucleocapsid protein from SARS-CoV-2, SARS-CoV, MERS and common cold viruses (OC43, HKU1, NL63, L229E) (Fig. 1). The peptides were additionally filtered to remove peptides having amino acids which could undergo any modifications. Peptides from the regions of nucleocapsid protein that were mutated in > 1% of sequenced cases in GISAID database were also excluded. In total, we selected three peptides as targets that could be reliably detected by mass spectrometry and were specific to SARS-CoV-2 as compared to other common coronaviruses. The selected peptides included ITFGGPSDSTGSNNQNGER, DGIIWVATEGALNTPK and NPANNAAIVLQLPQGTTLPK. Synthetic standards for these peptides were synthesized and characterized by vendor for the correct masses and were purified using high performance liquid chromatography. All the peptides were more than 95% pure and peptide stock concentration was determined by amino acid analysis.

### Evaluation of selected anti-peptide antibodies

**Fig. 2:**
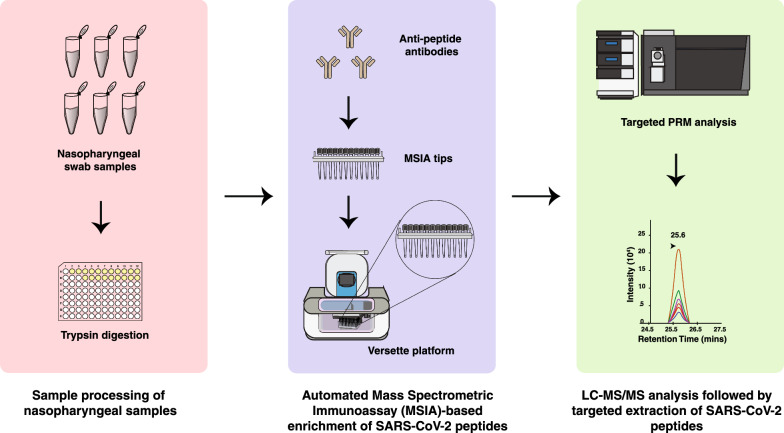
Experimental workflow for enrichment of nucleocapsid peptides using SISCAPA. Anti-peptide antibodies generated against three SARS-CoV-2 viral nucleocapsid protein-derived peptides (NPANNAAIVLQLPQGTTLPK, DGIIWVATEGALNTPK and ITFGGPSDSTGSNQNGER) were used to enrich from SARS-CoV-2 positive or negative nasopharyngeal swab samples. The anti-peptide antibodies were crosslinked to MSIA tips and peptides were enriched on MSIA tips using the Versette liquid handler platform as indicated. The enriched peptides were analyzed individually by targeted PRM analysis on an Orbitrap Eclipse mass spectrometer

Anti-peptide antibodies were generated for all the three selected peptides (SISCAPA Assay Technologies, Washington DC). As the nasopharyngeal swab samples were already tested for SARS-CoV-2 using RT-PCR-based molecular diagnostic testing, positive swab samples served as a positive control for evaluating the performance of the polyclonal antibodies and similarly negative swab samples were used as a negative control. Positive and negative samples were pooled separately and digested. The digests were then subjected for viral peptide enrichment on MSIA tips using Versette liquid handler platform. SISCAPA-based workflow employed for the detection of viral peptides is shown in Fig. 2. As expected, targeted analysis of the eluates from pooled negative swab samples did not show signal for any of the peptides analyzed, indicating high specificity of the enrichment and detection methods. In the pooled positive swab samples, we detected all the three peptides analyzed. These results indicate the feasibility of utilizing anti-peptides antibodies to detect viral peptides with high specificity.

### Determining peptide calibration curves and repeatability

**Fig. 3:**
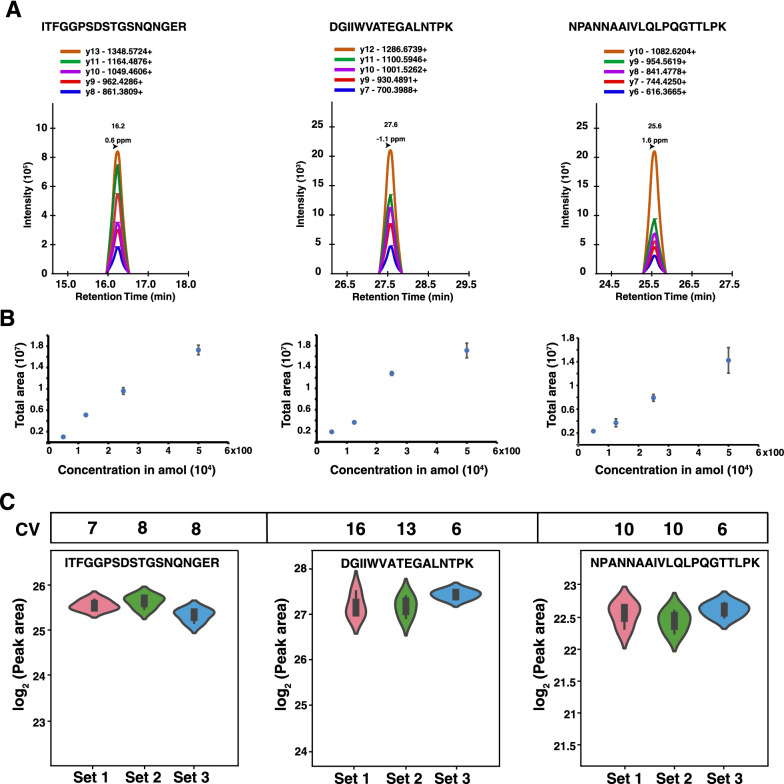
PRM analysis of viral nucleocapsid peptides after enrichment. **A** A representative figure of Skyline traces for NPANNAAIVLQLPQGTTLPK, DGIIWVATEGALNTPK and ITFGGPSDSTGSNQNGER peptides and their retention times. To determine the limit of detection (LOD), NPANNAAIVLQLPQGTTLPK, DGIIWVATEGALNTPK and ITFGGPSDSTGSNQNGER peptides were spiked into PBS and enrichment was done using the SISCAPA workflow. **B** Regression analysis presented in the figure demonstrates linearity of peak areas with the amount of spiked-in peptides as indicated. The transition ratios for selected fragment ions was reproducible regardless of amount of the analyte. Peak areas across all the peptide amounts spiked are shown in Additional file 1: Fig.S1. **C** The CVs calculated for three independent experiments for each peptide (each peptide analyzed in triplicate) are shown

To further characterize the sensitivity and reproducibility of the SISCAPA workflow, the limit of detection for the three peptides was determined by spiking different amounts of the synthetic light peptides (0, 50 amol, 125 amol, 250 amol, 500 amol, 1 fmol, 4 fmol, 20 fmol, 100 fmol) in phosphate buffered saline, followed by enrichment and PRM analysis. The list of transitions and their areas for all singly charged b and y ions were exported from Skyline. The coefficient of variation (CV) for each of the transitions was calculated for three replicates across all the dilutions and transitions with CV < 20 were considered for further analysis. The transitions and *m/z* considered for linear range and limit of detection (LOD) characterization are listed in Table 1 and Fig. 3A. All three peptides were detected at the lowest peptide amount injected i.e. 50 amol and displayed excellent linearity at lower concentrations (Fig. 3B). The CV was < 20 for all the three peptides analyzed. Next, we evaluated the reproducibility of the workflow by performing enrichment of peptides from the pooled SARS-CoV-2 positive nasopharyngeal swab digest in three different sets. In each experiment, three process replicates were used to measure the inter and intra-experiment CV. All the three peptides were reproducibly quantified with coefficient of variation of < 20 in both within the experiment and between the experiments (Fig. 3C).(see Table 2). These results indicate that the analytical workflow demonstrated in this study for the detection of SARS-CoV-2 is highly reproducible and can be deployed for analyzing clinical specimens.

**Table 1:** Nucleocapsid protein-derived peptides selected for SISCAPA assays and their transitions

Peptide	Position	*m/z*	Charge	Selected transitions
NPANNAAIVLQLPQGTTLPK	150–169	687.388	3	y10, y9, y8, y7, y6
DGIIWVATEGALNTPK	128–143	842.948	2	y12, y11, y10, y9, y7
ITFGGPSDSTGSNQNGER	15–32	912.411	2	y13, y11, y10, y9, y8

**Table 2:** Variability (reported as CV) for SISCAPA assay performed on 3 separate sets of pooled RT-PCR positive nasopharyngeal swab samples (Ct value < 24). The total area was considered for calculating the mean and standard deviation

	DGIIWVATEGALNTPK	ITFGGPSDSTGSNQNGER	NPANNAAIVLQLPQGTTLPK
Set 1			
Mean	1.64E + 08	4.90E + 07	6.04E + 06
SD	2.63E + 07	3.37E + 06	6.28E + 05
CV	16.04	6.88	10.38
Mean	1.62E + 08	5.15E + 07	5.69E + 06
Set 2			
SD	2.13E + 07	4.30E + 06	5.50E + 05
CV	13.14	8.34	9.66
Mean	1.87E + 08	4.28E + 07	6.27E + 06
Set 3			
SD	1.06E + 07	3.60E + 06	3.90E + 05
CV	5.64	8.41	6.21

### Detection of SARS-CoV-2 viral antigens from nasopharyngeal swab samples

**Fig. 4:**
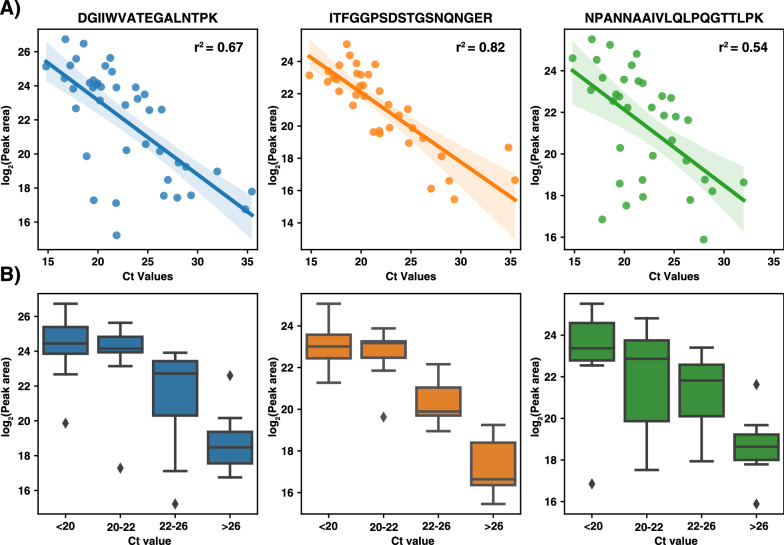
Detection of SARS-CoV-2 viral antigens from clinical samples using SISCAPA workflow. **A** 41 nasopharyngeal swab samples that were positive for SARS-CoV-2 using RT-PCR testing were processed using the workflow described in this study. The cumulative peak areas for all the three peptides are plotted against the Ct values reported by the RT-PCR testing. **B** Box plots of peak areas for each peptide are plotted across different groups based on the Ct values (Ct values < 20, 20–22, 22–26 and > 26) of the SARS-CoV-2 positive samples as indicated

Finally, we tested our approach on individual nasopharyngeal swab samples for viral detection. All the samples used in this study were tested for SARS-CoV-2 using routine RT-PCR diagnostic testing. Overall, we used 41 SARS-CoV-2 positive and 30 negative nasopharyngeal swab samples and performed enrichment of nucleocapsid peptides by anti-peptide antibodies using the workflow described in Fig. 2. The retention time for all the peptides was highly reproducible across the samples and we did not observe carryover in the negative samples. Overall, we did not detect PRM traces for the targeted peptides (ITFGGPSDSTGSNNQNGER, DGIIWVATEGALNTPK and NPANNAAIVLQLPQGTTLPK) in any of the 30 negative samples tested, indicating 100% specificity of the described workflow (Table 3). Among the RT-PCR positive samples tested for PRM analysis, samples were considered positive only if any two peptides were confidently identified with the expected transition ratios. Of the 41 samples, 38 samples were positive in PRM analysis with at least 2 peptides identified (all 3 peptides were identified in 30 samples). Quantitatively, each peptide was positively correlated with Ct value of the respective samples with a Pearson correlation of 0.82 for ITFGGPSDSTGSNNQNGER peptide, 0.67 for DGIIWVATEGALNTPK peptide and 0.54 for NPANNAAIVLQLPQGTTLPK peptide (Fig. 4A). To further assess the quantitative precision of the correlation with the Ct value, we categorized all the positive samples into 4 groups based on their Ct value ranges (Ct 16–20, 20–22, 22–26 and Ct > 26) as shown in Fig. 4B. Of the three peptides, ITFGGPSDSTGSNNQNGER peptide showed the best quantitative performance across all the four groups of Ct values. This is a pilot study to show that SISCAPA-based enrichment of viral peptides could indeed detect viral peptides from nasopharyngeal swab samples and also has the potential to detect SARS-CoV-2 from other clinical samples such as urine [27]. Overall, we employed a semi-automated approach for the detection of the viral peptides where automated steps included samples preparation in 96-well plates and immunoaffinity purification using an automatic liquid handler.

**Table 3:** Summary of MS-based detection of SARS-CoV-2 viral peptides from clinical nasopharyngeal swab samples using the SISCAPA workflow

	Detection of SARS-CoV-2 viral peptides by SISCAPA approach		
	Positive	Negative	
SARS-CoV-2 positive nasopharyngeal swab samples (n = 41)	38	3	Sensitivity-92.68%
SARS-CoV-2 negative nasopharyngeal swab samples (n = 30)	30	0	Specificity- ~ 100%

## Conclusions

In this study, we have demonstrated the applicability of anti-peptide antibodies for the enrichment of viral peptides from nasopharyngeal swab samples. This is the first study employing a SISCAPA approach for detection of novel SARS-CoV-2 in clinical samples. Using this approach, we found a specificity of 100% and sensitivity of ~ 93% for detection of novel SARS-CoV-2 in clinically tested nasopharyngeal swab samples. Employing monoclonal anti-peptide antibodies could further achieve higher sensitivity of detection especially in the samples with higher Ct value (low viral load). Also, optimization of chromatographic conditions such as run time and flow rates could help improve the throughput without compromising the sensitivity of detection of SARS-CoV-2. An additional advantage of this approach is that it can be applied to detection of viral peptides in samples such as formalin-fixed paraffin-embedded (FFPE) sections where enrichment of viral proteins (as opposed to peptides) is impossible.

## Supplementary Information

**Additional file 1:** **Figure S1.** Figure showing the correlation of peak areas across the entire range of synthetic peptide amounts spiked in phosphate buffered saline followed by enrichment and targeted analysis.

## Data Availability

The datasets generated during the current study are available under PRIDE repository with accession number PXD028505.

## Abbreviations

RT-PCR: Reverse transcriptase–polymerase chain reaction
SISCAPA: Stable Isotope Standards and Capture by Anti-Peptide Antibodies
PRM: Parallel reaction monitoring
NP swab: Nasopharyngeal swab
MSIA: Mass spectrometric immunoassay
LOD: Limit of detection
