# Correction: A SISCAPA-based approach for detection of SARS-CoV-2 viral antigens from clinical samples

**DOI:** 10.1186/s12014-022-09355-z

**Published:** 2022-05-04

**Authors:** Kiran K. Mangalaparthi, Sandip Chavan, Anil K. Madugundu, Santosh Renuse, Patrick M. Vanderboom, Anthony D. Maus, Jennifer Kemp, Benjamin R. Kipp, Stefan K. Grebe, Ravinder J. Singh, Akhilesh Pandey

**Affiliations:** 1 Department of Laboratory Medicine and Pathology, Division of Clinical Biochemistry and Immunology, Mayo Clinic, Rochester, MN 55905 USA; 2 Manipal Academy of Higher Education, Manipal, Karnataka 576104 India; 3 Institute of Bioinformatics, International Technology Park, Bangalore, Karnataka 560066 India; 4 Center for Molecular Medicine, National Institute of Mental Health and Neurosciences, Hosur Road, Bangalore, Karnataka 560029 India; 5 Center for Individualized Medicine, Mayo Clinic, Rochester, MN 55905 USA; 6 Department of Laboratory Medicine and Pathology, Division of Laboratory Genetics and Genomics, Mayo Clinic, Rochester, MN 55905 USA; 7 Department of Medicine, Division of Endocrinology, Mayo Clinic, Rochester, MN 55902 USA

## Correction to: Clin Proteom (2021) 18:25 https://doi.org/10.1186/s12014-021-09331-z

The original version [1] of this article contains errors in Tables 2 and 3 as follows:

In Table 2: Because of a formatting error, the mean values of three peptides in Set 2 were listed under Set 1 and the mean values for Set 3 were listed under Set 2.

In Table 3: The values under “Positive” and “Negative” columns for the row describing “SARS-CoV-2 negative nasopharyngeal swab samples (n = 30)” were switched and the “Specificity” was incorrectly represented as “~ 100%” instead of “100%.”

The authors regret these errors, which have now been corrected. The corrected Tables 2 and 3 are given.

**Table 2:** Variability (reported as CV) for SISCAPA assay performed on 3 separate sets of pooled RT-PCR positive nasopharyngeal swab samples (Ct value < 24)

	DGIIWVATEGALNTPK	ITFGGPSDSTGSNQNGER	NPANNAAIVLQLPQGTTLPK
Set 1			
Mean	1.64E+08	4.90E+07	6.04E+06
SD	2.63E+07	3.37E+06	6.28E+05
CV	16.04	6.88	10.38
Set 2			
Mean	1.62E+08	5.15E+07	5.69E+06
SD	2.13E+07	4.30E+06	5.50E+05
CV	13.14	8.34	9.66
Set 3			
Mean	1.87E+08	4.28E+07	6.27E+06
SD	1.06E+07	3.60E+06	3.90E+05
CV	5.64	8.41	6.21

The total area was considered for calculating the mean and standard deviation

**Table 3:** Summary of MS-based detection of SARS-CoV-2 viral peptides from clinical nasopharyngeal swab samples using the SISCAPA workflow

	Detection of SARS-CoV-2 viral peptides by SISCAPA approach		
	Positive	Negative	
SARS-CoV-2 positive nasopharyngeal swab samples (n = 41)	38	3	Sensitivity—92.68%
SARS-CoV-2 negative nasopharyngeal swab samples (n = 30)	0	30	Specificity—100%
