# Supplementary figures and images for: A SISCAPA-based approach for detection of SARS-CoV-2 viral antigens from clinical samples
Source: Clin Proteomics. 2021 Oct 22;18:25. doi: 10.1186/s12014-021-09331-z (PMC8532087; doi:10.1186/s12014-021-09331-z)

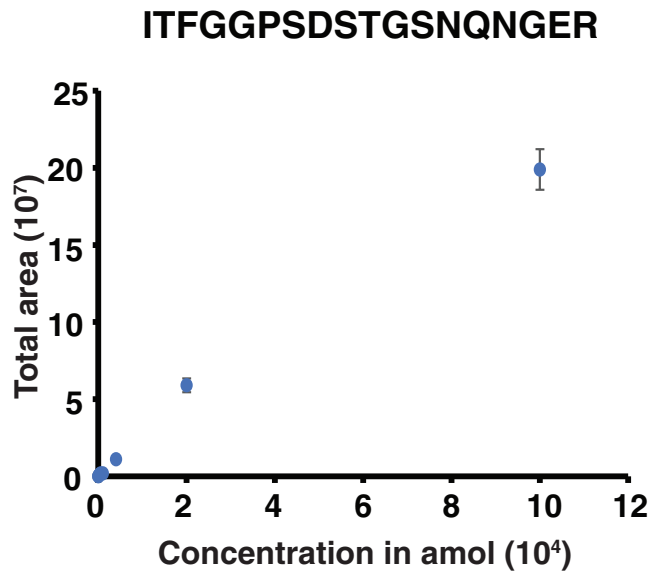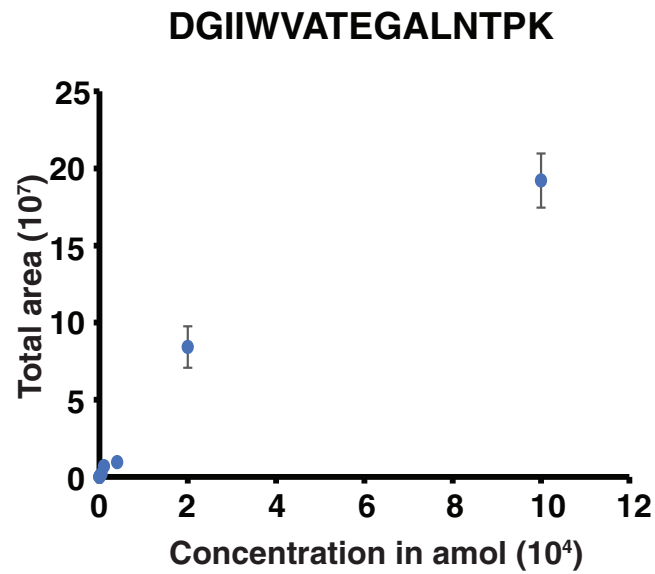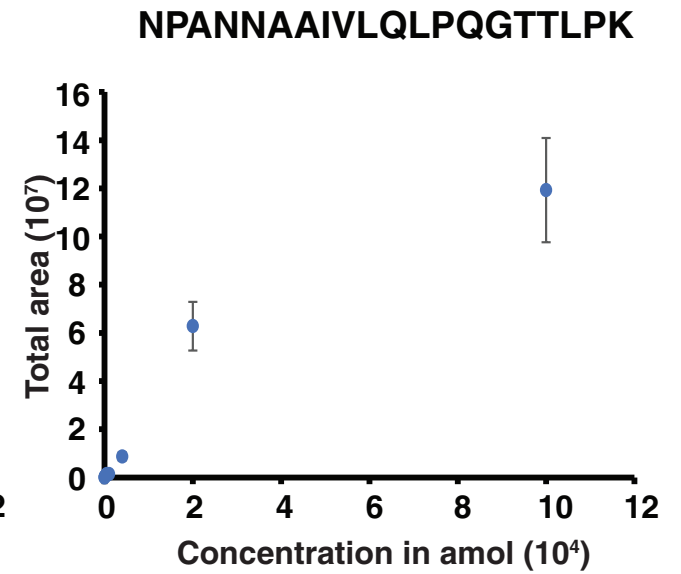

Supplement: Supplementary file 1 — Additional file 1: Figure S1. Figure showing the correlation of peak areas across the entire range of synthetic peptide amounts spiked in phosphate buffered saline followed by enrichment and targeted analysis. [file 12014_2021_9331_MOESM1_ESM.pdf]
